# Pain Acceptance as a Protective Factor Against Parental Stress in Parents with Chronic Pain Conditions

**DOI:** 10.3390/bs16050724

**Published:** 2026-05-08

**Authors:** Irene J. Muñoz-Peña, José L. González-Gutiérrez, Juan C. Pacho-Hernández, Laura Yunta-Rua, Almudena López-López

**Affiliations:** 1 Department of Psychology, Rey Juan Carlos University, Alcorcón, 28922 Madrid, Spain; irene.munoz@urjc.es (I.J.M.-P.); joseluis.gonzalez@urjc.es (J.L.G.-G.); laura.yunta@urjc.es (L.Y.-R.); 2 Department of Research and Psychology in Education, Universidad Complutense de Madrid, 28040 Madrid, Spain; jpacho@ucm.es

**Keywords:** pain acceptance, chronic pain, parenting, parenthood, parental stress, pain interference, pain intensity

## Abstract

Chronic pain can challenge effective parenting by limiting functional capacity and increasing vulnerability to stress. Within a process-based framework, pain acceptance—an element of psychological flexibility—may shape how parents manage pain-related demands during caregiving. This study examined associations between pain acceptance, pain intensity, pain interference in parenting, and parental stress in parents with chronic pain. A cross-sectional design was employed with 200 parents diagnosed with chronic pain. Participants completed the Chronic Pain Acceptance Questionnaire (CPAQ), the Parenting Tasks Interference Scale (PTI), and the Parental Stress Scale (PSS). Structural equation modeling (SEM) was conducted with robust estimation, and a serial mediation model (PROCESS Model 6) was used to estimate indirect effects using 5000 bootstrap resamples. Higher pain acceptance was associated with lower pain intensity and reduced interference in parenting. Pain interference, in turn, was related to higher parental stress, whereas pain intensity showed no direct association with stress. Serial mediation analyses showed a significant indirect association between pain acceptance and parental stress through pain interference, as well as a significant sequential indirect association involving pain intensity and pain interference. The indirect association through pain intensity alone was not significant. Model fit was excellent (CFI = 0.998; RMSEA = 0.021). The findings suggest that parental stress is more closely associated with pain acceptance and engagement in valued caregiving than with pain intensity. Process-based interventions targeting acceptance may be relevant for supporting family well-being.

## 1. Introduction

Although chronic pain is often perceived as a relatively uncommon condition, it is currently recognized as a significant public health issue, affecting approximately 20% of the global population. In Spain, various studies have estimated its prevalence to range between 17% and 25.9% in the general population (Dueñas et al., 2016, 2025; Mullins et al., 2022). Furthermore, epidemiological evidence indicates a marked gender disparity, with women exhibiting substantially higher prevalence rates than men (Dueñas et al., 2025; Jiménez-Trujillo et al., 2019). Chronic pain has a profound impact on individuals’ personal and psychosocial contexts, often leading to psychological distress and contributing to high morbidity rates worldwide (Treede et al., 2019; Vos et al., 2016). Its presence has been associated with significant impairments in individuals’ physical, social, and emotional functioning, resulting in greater disability and substantial restrictions in daily activities (Dueñas et al., 2025; Kawai et al., 2017). In addition, the often fluctuating and, at times, unpredictable nature of pain may pose significant challenges to fulfilling multiple social roles, including parenting responsibilities, increasing perceived stress (De Ruddere & Craig, 2016; Glette et al., 2020; Parton et al., 2022).

The processual model of stress developed by Lazarus and Folkman (1984) and Deater-Deckard’s (1998) parental stress model can help us better understand the interplay between stressors and perceived stress in parents with chronic pain. According to Lazarus and Folkman’s model, individuals’ appraisals of stressors determine how they allocate and mobilize available coping resources, and the coexistence of several stressors may increase vulnerability to stress and psychological distress. Extending this transactional view to the parenting context, Deater-Deckard’s (1998) parental stress model highlights that parental stress arises from a perceived imbalance between the demands of parenting and the resources available to meet them. Crucially, chronic pain may reduce actual coping resources (e.g., physical energy, emotional capacity), while also shaping parents’ appraisals of their ability to meet parenting demands, which together could influence to increase parental stress (Mun et al., 2017). Previous research supports these ideas by showing that the way parents manage chronic pain influences the amount of resources they have available to meet other demands (Maleki et al., 2012). Empirical evidence supports this notion, indicating that parents with chronic pain often rely on less-effective parenting coping strategies (Evans et al., 2005) and report more perceived stress related to parenting compared to their healthy counterparts (Muñoz-Peña et al., 2025), as well as to parents with other chronic conditions (e.g., multiple sclerosis, spinal cord injury, cancer) (Pastor-Bédard et al., 2023; Turney & Hardie, 2018).

From this perspective, the way pain is managed constitutes a key determinant of both an individual’s overall adaptation and the parental stress they experience. Within a transdiagnostic framework, increasing attention has been devoted in recent years to processes that facilitate better adaptation to chronic health conditions. One of the most relevant processes is experiential avoidance or, its counterpart, acceptance—a key component of the Psychological Flexibility Model (S. C. Hayes et al., 2006). Within a process-based psychology framework, pain acceptance is conceptualized as a core change process reflecting increased psychological flexibility. It involves shifting from maladaptive control-oriented strategies—such as attempts to suppress, avoid, or eliminate pain—to behavior patterns organized around personally valued actions. Rather than engaging in ineffective regulatory efforts, individuals allocate their psychological resources to actions that enhance meaning and vitality despite the continued presence of pain (McCracken et al., 2004a).

Through the lens of the stress process, pain acceptance can be conceptualized as a process that is activated in response to the stressful experience of pain. Rather than attempting to control or avoid the aversive experiences of pain, acceptance involves engaging with them while maintaining commitment to valued activities, facilitating adaptive cognitive appraisal and more effective allocation of personal resources. Indeed, pain acceptance has been linked to lower levels of perceived stress among individuals living with chronic pain (Costa & Pinto-Gouveia, 2011; Hann & McCracken, 2014). However, this association has not yet been specifically examined in the context of parental stress among parents living with chronic pain.

In addition to its association with perceived stress, research has consistently shown that greater pain acceptance is associated with lower perceived pain intensity (McCracken, 1998; Veehof et al., 2016; Ye et al., 2024) and reduced pain interference in daily activities among individuals living with chronic pain (Karayannis et al., 2023; Veehof et al., 2016; Vowles et al., 2008; Ye et al., 2024). To date, no studies have examined these associations in samples of parents with chronic pain, but based on the studies mentioned above, higher levels of pain acceptance are expected to be associated with lower levels of pain interference in caregiving tasks. Furthermore, given that pain intensity has been shown to directly influence the degree of interference experienced in daily life (Fayers et al., 2011), considering both constructs together is essential for understanding the impact of chronic pain on parenting.

Regarding the role of pain interference in the acceptance–stress interplay in parents, as previously noted, Deater-Deckard’s (1998) model conceptualizes parental stress as an imbalance between demands and resources. In the context of chronic pain, its interference in daily caregiving activities represents a concrete manifestation of this imbalance and may act as a mediator between pain and both perceived stress and psychological distress across different populations (Fragoso & McGonagle, 2018; Paz Domingo et al., 2017). From a processual perspective, when pain disrupts a parent’s ability to perform daily caregiving tasks, it signals a tangible constraint on available resources and heightens the perception of parenting-related stress. Therefore, pain interference would emerge as a key predictor of parental stress among parents experiencing chronic pain. Previous qualitative research has underscored the substantial difficulties individuals face in meeting parental responsibilities while managing chronic pain (Backman et al., 2007; Chew et al., 2019; Hadi et al., 2019; Kristiansen et al., 2012; Parton et al., 2022; Poh et al., 2017; Smith et al., 2011; Strunin & Boden, 2004). In addition, Evans et al. (2005) found that mothers with chronic pain reported greater interference with parenting activities compared to mothers without pain, and Smeele et al. (2020) found that mothers with rheumatoid arthritis experienced difficulties in performing postpartum parenting tasks. Although it is reasonable to assume a central role of this variable in the development and maintenance of parental stress, pain interference has not yet been systematically examined.

Taken together, these findings highlight the importance of examining not only the challenges that chronic pain imposes on parenting, but also the psychological processes that may buffer these effects. Applied to parenting, this framework suggests that parents who adopt an accepting stance toward pain are better able to allocate their limited resources flexibly, thereby reducing the impact of pain on caregiving responsibilities and, consequently, reducing perceived parental stress. Therefore, building on the theoretical and empirical frameworks described, the present study aimed to test a serial mediation model examining the pattern of associations between pain acceptance and parental stress in parents with chronic pain. Specifically, we proposed a model in which pain acceptance, pain intensity, pain interference in parenting tasks, and parental stress would be interrelated. This study is the first to examine this model in parents with chronic pain using structural equation modeling (SEM). Based on the previous literature, we hypothesized that (1) higher pain acceptance would be associated with lower pain intensity and less interference in parenting tasks, and (2) pain interference would mediate the relationship between pain acceptance and parental stress.

## 2. Materials and Methods

### 2.1. Participants

The study included 200 parents diagnosed with chronic pain from across Spain. Of these, 150 were women and 50 were men. Inclusion criteria were the following: being of legal adult age, having at least one child under 18 years of age, having a clinical diagnosis of chronic pain confirmed by a health professional, and having experienced symptoms for at least three months, in accordance with the latest guidelines of the International Association for the Study of Pain (IASP) (Treede et al., 2019). Exclusion criteria were as follows: psychotic symptoms or other major psychiatric disorders, severe chronic infectious, metabolic, renal, endocrine, oncological or neuromuscular diseases, concurrent participation in other research likely to influence the study variables, and cognitive or physical limitations that could interfere with informed consent or questionnaire completion. These criteria were applied to reduce potential confounding effects of complex medical conditions that may independently influence pain experience, pain interference in parenting tasks, and parental stress. Only cases considered severe according to medical criteria were excluded. This approach is consistent with common practices in chronic pain research, where comorbid medical conditions are frequently excluded to enhance sample homogeneity and internal validity (Salcedo et al., 2026).

Reported chronic pain conditions were varied: fibromyalgia was the most frequent (53%), followed by headache disorders (21.5%), inflammatory rheumatoid pain (13.5%), and back pain (8.5%). Less common conditions included osteoarthritis (1.5%), other musculoskeletal disorders (1%), and endometriosis (1%). Some participants reported more than one chronic pain condition.

Table 1 presents the sociodemographic characteristics of the sample. The sample had a mean age of 43.01 (SD = 6.38).

### 2.2. Measures and Instruments

#### 2.2.1. Sociodemographic Variables

Information collected included education level, employment status, marital status, and number of children. These data are presented in Table 1.

#### 2.2.2. Pain Acceptance

The Chronic Pain Acceptance Questionnaire (CPAQ) (McCracken et al., 2004b) was used to assess pain acceptance. This questionnaire consists of 20 Likert-type items with seven response options (from 0 = “never true” to 6 = “always true”). It is divided into two subscales: (1) engagement in activities (11 items), measuring participation in daily activities regardless of pain (e.g., “When my pain increases, I can still take care of my responsibilities”), and (2) openness to pain (9 items), assessing willingness to experience pain without resistance (e.g., “It’s not necessary for me to control my pain in order to handle my life well”). Scores range from 0 to 120, with higher scores indicating greater pain acceptance. The Spanish adaptation produced by González et al. (2010) was used in this study, showing good internal consistency for the full scale (α = 0.78) and for both subscales (α = 0.82 for engagement in activities and α = 0.78 for openness to pain).

#### 2.2.3. Pain Intensity

Pain intensity was assessed using a Visual Analogue Scale (VAS) (Scott & Huskisson, 1976), a validated, subjective measurement scale of acute and chronic pain (Delgado et al., 2018). The scale consists of a 10-point line representing the continuum of painful experience. Despite its simplicity, this instrument is robust, with satisfactory reliability (Price et al., 1983; Yarnitsky et al., 1996). In this study, pain intensity was measured with four items: (1) minimum intensity of pain during the last 15 days, (2) maximum intensity in the last 15 days, (3) usual pain intensity, and (4) pain intensity at the time of questionnaire completion. Responses were recorded on this scale, ranging from 1 (“no pain”) to 10 (“worst imaginable pain”). The total score was the sum of these four ratings. The internal consistency of the scale in the study sample was adequate (α = 0.81).

#### 2.2.4. Pain Interference in Parenting Tasks

The Parenting Tasks Index (PTI) assesses the impact of chronic illness or pain on daily parenting activities (e.g., feeding, children’s hygiene, or playing with the child) (Nehring & Cohen, 1995). The purpose of this scale is to evaluate the degree to which parents’ health affects their ability to perform daily tasks related to childcare. Three versions exist depending on the age of the oldest child: Infancy (birth to 1 year; e.g., “Breast or bottle feeding”), Childhood (1–10 years old; e.g., “Playing with your child outdoors”), and Adolescence (over 11 years old; e.g., “Assisting in child’s homework”). Participants completed the version corresponding to the age of their oldest child, as indicated in the questionnaire instructions. The scale offers four Likert-type response options (0 = no difficulty/no effort to 3 = severe difficulty/great effort). A total PTI score is obtained from the sum of the items. A higher total PTI score reflects greater impairment. The original test–retest reliability was acceptable (0.62–0.88) and internal consistency was excellent (0.93–0.96) (Nehring & Cohen, 1995). A Spanish translation of the original scale was used. The translation process followed the standard forward–backward translation method (Muñiz et al., 2013) to ensure linguistic and conceptual equivalence. First, the original items were translated into Spanish by a bilingual expert. Then, a separate bilingual translator, blind to the original version, performed a back-translation into the original language. Discrepancies were discussed and resolved to ensure semantic and conceptual equivalence. In this study, the term “chronic illness” used in the original items was adapted to “chronic pain” to ensure contextual relevance to the sample. Good indices of internal consistency were obtained for the translation of the three scales in the study sample (α = 0.88 for the infancy scale, α = 0.96 for the childhood scale and α = 0.93 for the adolescence scale).

#### 2.2.5. Parental Stress

Parental stress was assessed using the Parental Stress Scale (PSS) (Berry & Jones, 1995), which includes 18 items describing the parent–child relationship and perceptions of the parenting role. Using this scale, a parental stress score is calculated—both the positive aspects (e.g., “Having children gives me a more certain and optimistic view for the future”) and negative and stressful elements (e.g., “I feel overwhelmed by the responsibility of being a parent”). The original scale has adequate internal consistency (α = 0.84). The Spanish adaptation of the scale conducted by Oronoz et al. (2007), consisting of 12 Likert-type items with five response options (from 1 = strongly disagree to 5 = strongly agree), was used in this study. Internal consistency in this sample was adequate (α = 0.80).

### 2.3. Procedure

Prior to data collection, the project received approval from the University Research Ethics Committee. Recruitment combined online outreach (via patient organization channels, social media, and blogs targeting individuals with chronic) with offline methods including university bulletin boards and formal collaboration with national chronic pain associations. Associations agreeing to participate signed a collaboration agreement and disseminated the survey link through their preferred means (social networks, websites, or e-mail). A total of 36 organizations across Spain participated. Recruitment also employed snowball sampling, where enrolled parents could refer other eligible participants. Data were collected via an online platform using a cross-sectional design. The computerized assessment system required all items to be completed before submission; therefore, no missing data were present in the dataset. Before providing informed consent, participants were informed of the study’s purpose, data handling procedures, and their privacy rights.

### 2.4. Statistical Analysis

SPSS version 25.0 (IBM Corp., Armonk, NY, USA) and AMOS version 24.0 (IBM Corp., Armonk, NY, USA) were used for data analysis. Descriptive statistics were computed, including means and standard deviations, and bivariate correlations among variables were computed using SPSS.

**Figure 1:**
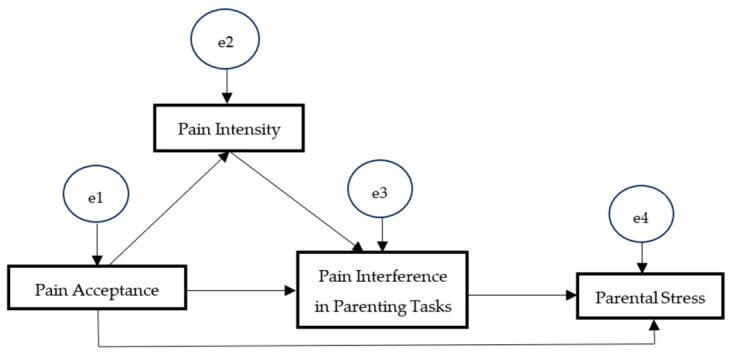
Research model.

The proposed mediation model (Figure 1) was tested using structural equation modeling (SEM) in AMOS. A path model with four observed variables was specified to examine the hypothesized relationships. An initial model including the direct path from pain acceptance to parental stress was first estimated, followed by a revised model excluding this path to evaluate model parsimony. The estimation procedure used was maximum likelihood (ML). Model fit was evaluated using the comparative fit index (CFI), goodness of fit index (GFI), Tucker–Lewis index (TLI), and root-mean-square error of approximation (RMSEA) (Jackson et al., 2009). For determining acceptable data fit, this study employed the criteria suggested by Kline (2015): CFI, GFI, and TLI values above 0.90 indicate that the model is acceptably fitted and an RMSEA value below 0.06 indicates close fitting. The chi-square goodness-of-fit was considered as a complementary measure, with a non-significant *p*-value indicating good fit.

While SEM was used to test the overall structural relationships and model fit, a serial mediation analysis was conducted using the PROCESS macro (model 6) with 5000 bootstrap resamples (A. F. Hayes, 2022). This approach was applied to estimate the significance and confidence intervals of the indirect effects, given that bootstrap procedures provide more robust estimates of indirect effects and are less dependent on normality assumptions (Preacher & Hayes, 2008). Bias-corrected 95% confidence intervals were used, and indirect effects were considered significant when the interval did not include zero. Given the relatively simple structure of the proposed model, which includes four observed variables, the sample size (*N* = 200) is in line with commonly accepted methodological recommendations for SEM and mediation analyses (Kline, 2015; Fritz & MacKinnon, 2007).

## 3. Results

### 3.1. Preliminary Analysis

Prior to model estimation, the assumptions of the regression analyses were tested. The first step was to explore the data, without using the estimated model, to determine if there were any univariate or multivariate outliers. The data were analyzed using a boxplot to identify univariate outliers and Mahalanobis distance test to identify multivariate outliers (critical value χ^2^(24) = 18.46, *p* < 0.001) (Tabachnick & Fidell, 2019). No extreme univariate or multivariate outliers were found. Multivariate normality was assessed using Mardia’s (1970) test of multivariate kurtosis. Results indicated that the assumption of multivariate normality was met (multivariate kurtosis = 1.426; critical ratio = 1.45).

Descriptive statistics indicated that pain acceptance had a mean of 49.29 (SD = 15.53), pain intensity had a mean of 24.55 (SD = 5.78), pain interference in parenting tasks had a mean of 36.14 (SD = 21.39), and parental stress had a mean of 29.59 (SD = 7.40). Regarding the correlations, pain acceptance demonstrated a significant negative association with pain intensity (r = −0.38, *p* < 0.01), as did pain acceptance with pain interference in parenting tasks (r = −0.42, *p* < 0.01) and pain acceptance with parental stress (r = −0.19, *p* < 0.01). Moreover, pain intensity was found to be positively correlated with pain interference in parenting tasks (r = 0.34, *p* < 0.01) but was not significantly associated with parental stress (r = 0.06, *p* > 0.05). Additionally, a positive correlation was observed between pain interference in parenting tasks and parental stress (r = 0.34, *p* < 0.01).

### 3.2. Testing the Proposed Model (SEM)

SEM was used to test the hypothesized relationships between the observed variables. First, the model including the direct path from pain acceptance to parental stress was estimated. The results indicated that this model demonstrated an acceptable fit to the data. The chi-square was non-significant (χ^2^ = 1.424; df = 1, *p* = 0.23). Considering the sensitivity of χ^2^ to the sample size, additional indices were considered (Medrano & Muñoz-Navarro, 2017). The comparative fit index (CFI) was 0.996, the normalized fit index (NFI) was 0.987, and the Tucker–Lewis index (TLI) was 0.976. The root-mean-square error of approximation (RMSEA) was 0.046, indicating a good model fit. However, the direct path from pain acceptance to parental stress was not statistically significant.

Given the non-significance of this direct path, a more parsimonious model excluding this pathway was subsequently estimated. This revised model showed an excellent fit to the data (χ^2^ = 2.174; df = 2, *p* = 0.34). The model yielded a CFI of 0.998, an NFI of 0.980, and a TLI of 0.995, all indicating excellent values. The RMSEA was 0.021, indicating close model fit. Compared to the initial model, the more parsimonious model demonstrated slightly improved fit indices.

**Figure 2:**
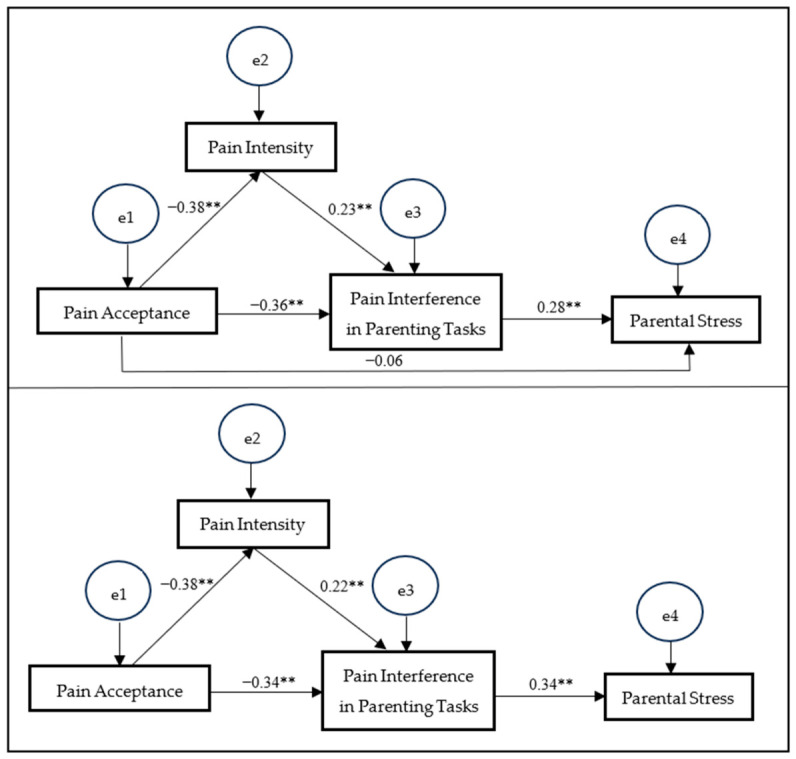
Serial mediation models including (**top**) and excluding (**bottom**) the direct path from pain acceptance to parental stress. The initial model includes the direct path, whereas the final model excludes the non-significant path. Standardized regression coefficients (β) are presented. ** *p* < 0.001.

For clarity, Figure 2 presents both the initial model (including the direct path from pain acceptance to parental stress) and the final model (excluding the non-significant direct path). In the final model, all estimated path coefficients were statistically significant, providing support for the hypothesized associations.

### 3.3. Serial Mediation Analysis

**Figure 3:**
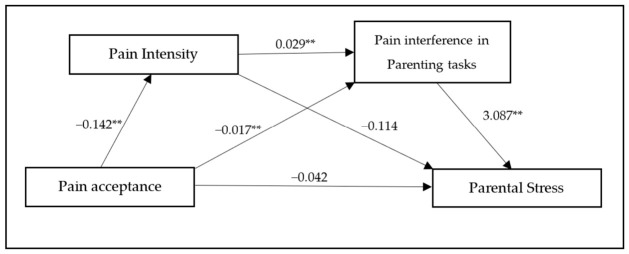
Path analysis of the serial mediation model. Note: ** *p* < 0.001.

To evaluate the significance of indirect effects, a serial mediation analysis was conducted using PROCESS for SPSS with 5000 bootstrap resamples, following A. F. Hayes (2022). Pain intensity and pain interference were specified as sequential mediators of the relationship between pain acceptance and parental stress. The results are presented in Table 2 and Table 3 and illustrated in Figure 3.

As shown in Table 2, pain acceptance significantly and negatively predicted pain intensity (*B* = −0.142, *p* < 0.001) and pain interference in parenting tasks (*B* = −0.017, *p* < 0.001). Furthermore, pain intensity significantly and positively predicted pain interference (*B* = 0.029, *p* < 0.001).

Regarding the final outcome, pain interference was a strong positive predictor of parental stress (*B* = 3.087, *p* < 0.001). In contrast, pain intensity did not significantly predict parental stress (*B* = −0.114, *p* = 0.237). The direct effect of pain acceptance on parental stress was not significant when mediators were included in the model (*B* = −0.042, *p* = 0.260).

The results of the mediation analysis are presented in Table 3. The total effect of pain acceptance on parental stress was significant (B = −0.091, 95% CI [−0.156, −0.025]). The total indirect effect through the proposed mediators was also significant (B = −0.049, 95% CI [−0.096, −0.007]). Specifically, the indirect effect of pain acceptance on parental stress through pain interference was significant (95% CI [−0.092, −0.024]), whereas the indirect effect through pain intensity alone was not significant (95% CI [−0.010, 0.054]). The hypothesized serial mediating effect through both pain intensity and pain interference was statistically significant (95% CI [−0.032, −0.004]). These results support the presence of a significant sequential mediation pathway.

## 4. Discussion

Guided by stress and coping theories and the psychological flexibility framework, this study aimed to examine the relationship between pain acceptance and parental stress among parents with chronic pain and the role of pain intensity and pain interference in this relationship. In line with our expectations, the data are consistent with a model in which higher pain acceptance, lower parental stress, and pain interference in parenting tasks are interrelated. In addition, the data are statistically consistent with a pattern of associations in which pain acceptance, pain interference, and pain intensity are related to one another in a way consistent with a mediation model. These findings underscore pain acceptance as an important psychological resource for parents living with chronic pain, with associations extending to both overall wellbeing and pain-related and parenting-related outcomes. Moreover, our data are consistent with a central position of pain interference within the network of associations related to parental stress.

The study of psychological processes underlying parental stress in individuals with chronic pain—and the predictors of pain interference in parental tasks—is very scarce, despite several studies documenting the functional impact of chronic pain on the caregiving role. For example, Evans et al. (2005) showed that mothers with chronic pain reported greater disruption in parenting activities, measured by the interference in the parenting tasks questionnaire (PTI), compared to pain-free mothers. Smeele et al. (2020) reported that mothers with chronic pain experienced notable difficulties completing routine physical childcare tasks due to pain and fatigue, highlighting the functional impact of pain on parenting. More recent research also points to the chronic nature of parental stress in the context of pain (Beveridge et al., 2024) and the relevance of examining parenting-specific interference in families affected by chronic pain (Evans, 2004; Parton et al., 2022).

As stated above, our mediation analyses were consistent with a pattern in which higher acceptance, lower parental stress, and pain interference were interrelated. To our knowledge, no prior studies have examined pain acceptance in the parenting context, while this pattern aligns with previous findings in individuals with chronic pain. Some studies have shown that greater pain acceptance is associated with lower emotional stress and better psychological adjustment among individuals with chronic pain (Costa & Pinto-Gouveia, 2011; Hann & McCracken, 2014). In addition, the predictive role of pain acceptance in relation to interference in daily activities has been documented, with higher acceptance related to lower pain interference in basic and instrumental activities of daily living (Karayannis et al., 2023; Vowles & McCracken, 2008).

Consistent with Deater-Deckard’s (1998) conceptualization of parental stress and the transactional model of stress proposed by Lazarus and Folkman (1984), pain interference can be understood as a behavioral manifestation of an imbalance between caregiving demands and perceived coping resources, which may reflect the inadequacy of appraisal and contribute to higher perceived stress. Our results are consistent with this framework and with previous research involving parents with rheumatoid arthritis, which found that the degree to which pain and other illness symptoms made parenting activities more difficult was associated with parental stress and depressive symptoms (Katz et al., 2003; Zelkowitz et al., 2013). Beyond this, the finding that the association between pain acceptance and parental stress became non-significant after accounting for pain interference is consistent with the possibility that pain interference may play a key role in the pattern of associations linking acceptance and parental stress. In line with the Psychological Flexibility Model (S. C. Hayes et al., 2006), although no causal inferences can be drawn from the present data, this pattern of associations highlights the potential relevance of examining how acceptance may be reflected in sustained engagement in value-consistent caregiving behaviors.

In this context, lower pain interference in parenting tasks could reflect not only a better balance between caregiving demands and coping capacity (in line with Deater-Deckard’s processual model of parental stress) but also a process of committed action related to caregiving values. From the perspective of psychological flexibility (S. C. Hayes et al., 2006), the acceptance process has been associated with lower reliance on avoidance-oriented responses and facilitates engagement in valued activities—a process that has been linked to lower functional interference in daily life (Karayannis et al., 2023). Our findings are consistent with the idea that these processes may also be relevant within the parenting domain, whereby higher acceptance is associated with less disruption of pain in caregiving tasks. From a theoretical perspective, both the willingness to experience pain as a natural part of life and the disposition to engage in activities despite pain may support more flexible responses to parenting demands in the context of ongoing pain. Within this framework, it is plausible that acceptance is linked to greater engagement in valued caregiving behaviors, which may, in turn, be more directly associated with parental stress. However, this interpretation remains speculative given the cross-sectional nature of the data. The proposed model included not only the existence of a direct relationship between pain acceptance and pain interference but also incorporated pain intensity within the model as a variable linking acceptance and pain-related interference in parental caregiving. Our data are consistent with a pattern of associations compatible with a sequential mediation process, although no causal inferences can be drawn from the present cross-sectional design. The predictive role of pain acceptance in relation to pain intensity has been examined in the previous literature on chronic pain populations, showing that greater acceptance is linked to lower perceived pain levels (McCracken, 1998; Veehof et al., 2016; Ye et al., 2024). Recent evidence from patients with rheumatoid arthritis also supports this pattern: higher pain acceptance has been associated with lower pain intensity and better illness adjustment, further reinforcing the robustness of this association across chronic pain conditions (Akça Doğan et al., 2024). Our results extend the knowledge about these relationships to the parenting context, as no previous studies have specifically examined this relationship among parents with chronic pain.

Importantly, the absence of a direct association between pain intensity and parental stress—together with the role of pain interference as a linking variable—suggests that it may not be the nociceptive input per se that is most closely related to parental distress, but rather the way in which the pain experience is managed and the extent to which it is associated with disruptions in valued caregiving activities. In other words, from a theoretical standpoint, the magnitude or quality of the aversive stimulus appears less relevant for understanding perceived stress than the individual’s capacity to sustain active engagement in caregiving roles despite pain, as reflected in lower levels of pain interference in parental caregiving activities. These results are consistent with the previous literature in different chronic pain populations showing the mediating role of pain interference in the relationship between pain intensity and emotional distress, in which pain interference in daily life emerges as a stronger prediction of depression than pain severity (Li et al., 2022; López-Lopez et al., 2014; Villela et al., 2025).

Taken together, these findings are consistent with an account in which acceptance is primarily associated with lower levels of parenting-specific interference rather than with direct reductions in stress, and they contribute to the literature in several ways. First, they provide quantitative evidence for the role of pain interference in parenting tasks in understanding how the impact of chronic pain relates to elevated parental stress, thereby extending previous qualitative findings (Backman et al., 2007; Chew et al., 2019; Parton et al., 2022). Second, they highlight pain acceptance as a potentially modifiable psychological factor that may be linked to lower stress, consistent with the Psychological Flexibility Model (S. C. Hayes et al., 2006). Finally, this study addresses an important research gap by including both mothers and fathers. These findings have several clinical implications. First, they highlight the relevance of interventions that target acceptance processes. Interventions for parents living with chronic pain may benefit from integrating acceptance-enhancing strategies with explicit training in committed, values-based action embedded within caregiving routines (e.g., values clarification around parenting roles, graded exposure to pain-related sensations during childcare tasks, activity pacing, and problem-solving to sustain engagement under symptom fluctuations). Acceptance and Commitment Therapy (ACT) has produced substantial evidence for reducing pain interference and improving psychological flexibility in adults with chronic pain (McCracken & Vowles, 2014; Vowles & McCracken, 2008). Moreover, ACT-informed parenting programs developed for families facing chronic health conditions in their children have demonstrated reductions in parental stress and improvements in parental well-being (Brown et al., 2014; Whittingham et al., 2016). Although these interventions do not target parents with chronic pain, they illustrate that acceptance-based approaches can be successfully adapted to caregiving contexts and may therefore offer a promising framework for supporting parents who experience pain-related disruptions in daily parenting tasks. A practical adaptation of Acceptance and Commitment Therapy (ACT) for this population might involve helping parents recognize and disengage from pain-related negative thoughts, clarify their parenting values, and identify caregiving tasks they can continue to perform despite pain, as well as those they find more difficult. In cases where pain considerably limits specific tasks, exploring alternative strategies, such as delegating responsibilities to other family members, may help reduce pain interference in parenting, which was identified in this study as a key process within the observed pattern of associations. Incorporating parenting-focused ACT exercises (e.g., values-based caregiving practice, mindfulness during routine childcare activities, or strategies to respond flexibly to pain while performing parenting tasks) may further strengthen the intervention’s effects and enhance its ecological validity.

A key strength of this study lies in its relatively large sample of parents with chronic pain and its focus on parenting-specific outcomes. The use of structural equation modeling and bootstrapping provides statistically rigorous support for the hypothesized mediation pathways. Nonetheless, several limitations should be noted. The cross-sectional design precludes causal inferences, and future longitudinal studies are needed to establish temporal ordering. The reliance on self-report measures may have introduced shared-method variance, and the predominance of mothers in the sample limits generalizability to fathers. However, this imbalance may partly reflect the higher prevalence of chronic pain among women in Spain (Dueñas et al., 2025; Jiménez-Trujillo et al., 2019). In addition, the exclusion of participants with severe comorbid medical conditions may limit the generalizability of the findings, as chronic pain often co-occurs with other health conditions. Recruitment through patient associations and online platforms may have introduced selection bias.

Future research should replicate these findings in more diverse samples, including more fathers and underrepresented chronic pain conditions. It would also be useful to account for child-related factors, such as the child’s age, as previous research suggests that illness-related interference in parenting tends to decrease as children become more independent (Mukherjee, 2005). In addition, examining the role of social support may offer further insight, as lower support has been associated with greater difficulty performing parenting tasks and higher psychological distress in parents facing chronic illness (Mukherjee, 2005). In this regard, exploring potential differences between single-parent and two-parent households may be particularly relevant, as research indicates that parents in single-parent families may experience lower levels of perceived social support and greater challenges in parenting practices and stress regulation compared to partnered parents (Hosokawa & Katsura, 2024). Thus, examining family structure in parents with chronic pain may provide further insight into contextual factors associated with parenting-related outcomes. Future work should test these pathways using longitudinal and experimental designs to establish temporal precedence—examining whether session-to-session gains in acceptance prospectively predict subsequent reductions in interference and, in turn, stress, above and beyond pain intensity. Incorporating an ecological momentary assessment of parenting demands and interference would strengthen causal inference and ecological validity, while probing moderators (e.g., child age, co-parent support, fatigue) could identify for whom these processes are most potent. In addition, future research should consider clinical trials aimed at enhancing pain acceptance in parents with chronic pain, testing whether increases in acceptance are prospectively associated with subsequent reductions in pain interference in parenting tasks and, ultimately, parental stress. It would also be valuable to examine additional components of psychological flexibility beyond acceptance, such as cognitive defusion or valued-based action, as well as to further validate parenting-specific measures of pain interference as potential protective factors in the parenting context.

## 5. Conclusions

Overall, this study provides novel evidence consistent with the view that pain acceptance is an important psychological resource associated with lower parental stress among parents with chronic pain. The findings suggest that acceptance may be more closely linked to parental stress through its association with lower levels of functional disruption, while pain intensity appears to play a more indirect role. From a clinical perspective, these results underscore the clinical relevance of fostering acceptance-based and value-consistent action in interventions for parents with chronic pain. More broadly, they point to the importance of considering both psychological flexibility processes and parenting-specific functional outcomes in future clinical and research efforts aimed at supporting families affected by chronic pain.

## Figures and Tables

**Table 1:** Sociodemographic characteristics of the sample.

Participants (*n* = 200)		
Educational level		
	Primary studies	15 (7.5%)
	Secondary studies	28 (14%)
	Intermediate education	71 (35.5%)
	Higher education	71 (35.5%)
	Postgraduate studies	15 (7.5%)
Employment status		
	Employed	135 (67.5%)
	Unemployed	65 (32.5%)
Marital status		
	Married	156 (78%)
	In a relationship	16 (8%)
	Single	6 (3%)
	Separated/Divorced	22 (11%)
Number of children		
	One	74 (37%)
	Two	104 (52%)
	Three	19 (9.5%)
	Four	3 (1.5%)

Note. *n* = number of participants.

**Table 2:** Regression results of the serial mediation model.

Outcome Variable	Predictor	B	SE	t	*p*	R^2^
Pain intensity	Pain acceptance	−0.142	0.025	−5.8162	<0.001	0.146
PIIPT	Pain acceptance	−0.017	0.003	−5.3834	<0.001	0.247
	Pain intensity	0.029	0.009	3.4543	<0.001	
Parental Stress	Pain acceptance	−0.042	0.037	−1.1295	0.260	0.107
	Pain intensity	−0.114	0.096	−1.1864	0.237	
	PIIPT	3.087	0.788	3.9176	<0.001	

Note. B: Unstandardized regression coefficient; SE: standard error; PIIPT: Pain Interference in Parenting Tasks.

**Table 3:** Analysis of mediating effects between variables.

Path	Effect	BootSE	LLCI	ULCI
Indirect effects				
Pain Acceptance → Pain Intensity → Parental Stress	0.016	0.016	−0.010	0.054
Pain Acceptance → PIIPT → Parental Stress	−0.052	0.017	−0.092	−0.024
Pain Acceptance → Pain Intensity → PIIPT → Parental Stress	−0.013	0.007	−0.032	−0.004
Total indirect effect	−0.049	0.023	−0.096	−0.007
Direct effect Pain acceptance → Parental Stress	−0.042	0.037	−0.116	0.031
Total effect Pain Acceptance → Parental Stress	−0.091	0.033	−0.156	−0.025

Note. BootSE: bootstrap standard error; LLCI: lower level of the 95% confidence interval; ULCI: upper level of the 95% confidence interval; PIIPT: Pain interference in parenting tasks. Confidence intervals for indirect effects were calculated using 5000 bootstrap samples. Indirect effects are considered statistically significant if the 95% confidence interval does not include zero.

## Data Availability

The data and the forms used in this study to collect information from participants can be found on the Open Science Framework (OSF): https://osf.io/4qwx3 (accessed on 1 May 2026).
